# Mixed Insanity—Reason and Madness
*Un Chapitre Oublié de la Pathologie Mentale. Par le Docteur Moreau, Médecin de l'Hospice de Bicêtre. Paris, 1850.


**Published:** 1850-10-01

**Authors:** 


### Art. IV.—MIXED INSANITY—REASON AND MADNESS.*

Dr. Moreau, the eminent physician of the Bicêtre, has just published an interesting brochure, which he entitles, "*Un Chapitre Oublié de la Pathologie Mentale;*" and although put forth in an unpretending form, we have no hesitation in pronouncing it a valuable contribution to psychological literature.

Dr. Moreau commences his observations by calling our attention to a form of insanity which is very remarkable, and which we have frequently observed in our own practice; nevertheless, it has not, we believe, been sufficiently or clearly described. We allude to that peculiar *state of mind in which reason and a certain degree of madness not only co-exist, but appear to be almost inseparably blended.*

* Un Chapitre Oublié de la Pathologie Mentale. Par le Docteur Moreau, Médecin de l'Hospice de Bicêtre. Paris, 1850.

The proposition of Dr. Moreau is given in the following words:—

"Madness and reason are two extreme terms of mental dynanism, which in some cases so closely approximate, and become so confounded with each other, as to give rise to a separate state of intellect which participates in reason and madness at the same time. The human mind," he observes, "sometimes undergoes modifications so peculiar in their nature that we are obliged to pronounce the most contradictory opinions respecting such persons;—we praise their abilities, and extol their genius; nevertheless, we cannot escape the conviction that we are dealing, in certain respects, at least with minds that are not only capricious and eccentric, but *positively deranged.* To such individuals we may apply the words of an English author—'They certainly are cracked, but the crack lets in the light.'

"It is generally supposed that between madness and reason there exists a well-defined line of demarcation, and that the one condition necessarily excludes the other; which certainly is the case when we speak of madness properly so called—that is, when its symptoms are fully declared; but it is very much otherwise, and the question becomes difficult to resolve, when we have to deal with those peculiar modifications of the mind in which there appears to be a *mélange* between madness and reason, a sort of '*mixed state,*' resulting from psychological conditions peculiar to both these states. Those who have made mental pathology their study, already agree in recognising a sort of admixture between madness and reason in such cases as the following—I. An individual who is deranged in his actions, thoughts, and sensations, nevertheless entertains a consciousness of his aberration—he appreciates his state; he feels the malady growing upon him, and struggles against its fatal influence. II. Another individual, by virtue of some peculiar law of his intellectual organism, attaches himself to a single idea, and becomes in every sense a monomaniac, although on all other subjects save the one of his aberration, he is perfectly rational.

"These two pathological states of the mind, however, differ essentially from the one under consideration, inasmuch as there is in neither of them, properly speaking, a fusion between the normal and abnormal state of the intellect; in reality, there exists only in such cases a sort of co-existence—a juxtaposition between madness and reason, both being essentially distinct, although occurring in the same person. In the first case, we observe a certain duality of thought and feeling—hence, at the same time that the individual is irrational, he is rational; a host of incoherent thoughts crowd upon him, which he expresses in language and in extravagant acts; and when he feels himself overcome by his erroneous apprehensions, and vain terrors, he is fully conscious of his inability to shake them off. Here, then, we have evidently two distinct beings in the same person; the *homo duplex* complete—the unity of the ME (the *ego*) is destroyed. In the second case, the distinction between the two individualities is more self-evident; for it is certain that beyond the immediate circle of his erroneous conception, the monomaniac preserves his natural reason; nor is it possible beyond this to discover any difference between him and individuals reputed to be of sound mind.

The monomaniac, in point of fact, exercises his mental faculties in two different spheres—in the one he is insane, in the other he is rational.

"Let us now proceed to give a general idea of that particular mental state which it is the object of this memoir to elucidate, which is one of those psychological conditions that may easily be proved, however difficult to describe. Upon the distinction between our intellectual powers and faculties little need be said; but it may be observed, that although the phenomena of the mind have been scrupulously analysed, no person has yet been able to circumscribe its limits, or calculate the innumerable forms which its activity must assume, according to the infinite diversity of different idiosyncrasies. Through what variety of modifications must it pass before breaking through the bounds assigned to its normal state of existence?—before entering into a sphere of activity entirely new—that of an interior life—a state of dream, delirium, or madness? Certain it is, that under a variety of psychological and moral conditions—more particularly hereditary predisposition, its constitution will become so modified as to exhibit all the indications of being in a state of rationality, while it is at the same time in a state of madness. In such cases there is no longer a question, as in the preceding examples, of a co-existence between the sane and insane state; there is here a positive fusion between them; and this peculiar lesion of intellect, although difficult to describe in the language of the schools, is open to the clearest diagnosis.

"This form of insanity, which I have designated the *Etat Mixte*, has a twofold origin; it may arise from direct hereditary transmission, or from special peculiarities in the constitution. The law of hereditary transmission manifests itself so clearly, that it has never been disputed, in so far as the principles of material organization may be concerned; but some authors have denied its application to more subtile phenomena. They are obviously in error; for experience most incontestably proves that intellectual phenomena by no means escape the common hereditary laws of our nature. Besides, why should it be otherwise? a peculiar system of organs is indispensable to the manifestation of the immaterial principle, and why should the encephalic and nervous systems be exempted from this hereditary train of causation more than any other organs of the body performing functions more obviously material? Rest assured that the law of hereditary transmission has impressed its seal upon all forms of mental dynanism—on all modes of the manifestation of the thinking principle; from the most elementary to the most transcendental; from the most distant to the nearest points of material contact. Peculiarities affecting general sensibility—the external senses of touch, smell, sight, hearing, are well known to be transmitted from family to family—nay, from generation to generation. And why should not the intellectual faculties be under the immediate influence of analogous laws? We see the same type reproduced constantly in the members of the same family—the children of which inherit the tastes, propensities, and passions of their parents. Such facts pass constantly unobserved; until some moral outrage on society calls our attention to the history of particular individuals. Among these propensities which are most obviously transmitted from parent to child—from family to family—we may specify in particular, with Dr. Lucas—the propensities to drink, the passion of gambling, and, above all, excessive sexual indulgence; in addition to which, facts abundantly prove the hereditary predisposition to the commission of certain crimes, both against the person and against property.

"One of the most curious facts," says Dr. Moreau, "which cannot be too attentively considered, is this, that we have seen the madness of children assume precisely the same characters, and all the same peculiarities, as the madness of their parents. All authors report such cases. Many children who become insane exhibit the same intellectual anomalies, and become affected by the same causes—nay, even at the same age as their father or their mother. Attempts have been made to explain this fact, sometimes by ascribing it to moral causes; sometimes by referring it to peculiarities of physical organization; but whichever theory be adopted, the hereditary repetition of the same phenomena assuming an identity in manifestation is beyond a doubt. 'Hereditary mania,' says Esquirol, 'appears at the same age in the child as it did in the father; it is superinduced by the same causes, and it assumes the same character. A Swiss merchant saw two of his sons die insane, each being nineteen years of age. A lady became insane twenty-five years after her accouchement: in like manner, her daughter became deranged twenty-five years after her accouchement. In one family, the father, son, and grandson, all committed suicide about the fiftieth year of their respective ages. We had at the Salpêtrière an unfortunate *fille publique* who threw herself three times in the river after her orgies; and her sister killed herself after drinking wine. A gentleman, overpowered by the first horrible events of the revolution, became melancholy, and shut himself up in his apartment, where he confined himself for ten years; his daughter, about the same age, fell into the same state, and in like manner, she refused to quit her room. This predisposition,' observes Esquirol, 'manifesting itself by peculiar signs, in accordance with the moral and intellectual character of individuals, is no more surprising in respect to insanity than in respect to gout, consumption, or any other physical disease. Other facts might be added. Madame W——, an inmate at Charenton, imagined that the whole world had conspired to poison her; her mother long laboured under a similar delusion. Madame D—— had eight of her family insane—her father, two sisters, two brothers, two cousins, and an aunt. Monsieur C——, after having resisted for many months a strong propensity to commit suicide, at length blew out his brains; he could never pass along the sides of a river or near a well without experiencing the strongest temptation to commit the act. His eldest sister, after being for many years haunted by the same ideas as her brother, committed suicide; she dared not pass over a bridge without being accompanied by some one to protect her. Mademoiselle B—— three times attempted to destroy herself: once by endeavouring to throw herself into a well, and twice by attempting to hang herself. Her mother, in a similar state of mental derangement, attempted to destroy herself by the same means, which she resorted to in the same succession. Mademoiselle H——, among other delusions, fancied that Charles X. was in love with her; and that she, in return, had vowed a tender and eternal attachment to his majesty. Her passion knew neither bounds nor fear; she contrived to escape the notice of the guards at the Tuileries, and found her way into the apartments of the king. The poor lady was immediately seized and sent, under the protection of her elder sister, to Charenton. Some time afterwards, this same sister was admitted, insane, into the Charenton, her delusion having assumed precisely the same character.'

"Two sisters had been suffering under monomania, many years before they were admitted into the same hospital. However whimsical or extravagant they happened to be, their fancies always assimilated. They imagined that all their thoughts and actions were influenced and governed by electricity, and that Monsieur Duplafon was so all-powerful that it was necessary to consult him concerning all their affairs. A mother and daughter, Mesdames B——, both imagined themselves to be under the special protection of spirits they called '*the Airs*.' A young melancholic lady, when asked her name, invariably answered, '*l'Inconnu;*' her brother, who was also melancholic, entertained the same fancy, and was always much enraged if called by any other name than '*l'Inconnu*.' Madame de B—— supposed that she had created a fantastic being to have dominion over her, whom she called '*Solomon;*' he was her evil genius, and she complained bitterly of the torments he made her suffer. Her father, under an analogous delusion, complained of the sufferings he endured through the agency of a malicious sylph, whom he designated '*Stratageme*.' The following facts were related to me by Esquirol:— 'Three brothers, successively, and within a few years of each other, committed suicide. There remained only a fourth brother and one sister. A brilliant fortune, high personal accomplishments, the tenderness and devotion of a wife he tenderly adored, with three children of great promise, assured, or might have assured, for Monsieur M—— a happy position and an honourable rank in the world. Might it not be supposed that with such cheering prospects before him he would have uprooted from his breast the germs of that propensity which had urged his three unhappy brothers to commit suicide? Alas! more miserable than they, he felt the hereditary evil preying upon his heart; and coming one morning to Monsieur Esquirol, he said to him, with the greatest calmness—"I cannot get rid of the darkest presentiments; I feel that I ought to end my life as my brothers did theirs; I am harassed by ideas which will carry me away in spite of myself, and the care and solicitude of my dear wife." A short time afterwards he committed suicide. His sister succumbed to the same fatality; she also destroyed herself.'

"So strong, then, is the influence of hereditary transmission, that a similitude may be observed to pervade a whole series of aberrant intellectual acts; and considering the very numerous facts which establish such a conformity in hereditary organization, can we deviate from the path of rigorous induction in admitting that among such individuals there exists, in all probability, a peculiar *cerebro-mental* structure rather than any anomaly of functions not yet developed? When we see an individual differing from other men generally, as respects his manners, habits, character, judgment, eccentricity of ideas, tendency to push beyond all bounds the affective and intellectual attributes of his nature, disdaining realities and attaching himself to ideal and fantastic notions, are we not justified in referring these strange and extravagant manifestations of intellect to some original irregularities of organization?"

Having called our attention to the uniformity of type which, as in the above remarkable cases, may often be observed in the character of insanity when developed under direct hereditary causes, Dr. Moreau proceeds to point out that we are not, in all cases, to expect that the uniformity of these phenomena—the repetition, as it were, of their identity, shall be equally striking. They have their different shades of intensity, their modifications, and degrees.

"And here we come to that *mélange* between madness and reason—that *état mixte*—which it is our more immediate object to discuss. Viewing the disease under its hereditary aspect, we may easily conceive that the offspring of the insane, without being positively deranged, may nevertheless manifest certain moral peculiarities resembling, more or less, the malady of their parents; and this, be it observed, is unquestionably the case in many diseases which are *purement physique.* Thus a scrofulous father may not give birth to children as scrofulous as himself; but it often happens that the constitution of some of his offspring will be sufficiently infected to indicate the hereditary taint. We observe this in consumption, gout, and all other hereditary diseases. So, also, the intellect, before being sufficiently deranged to be thrown out of its apparently normal limits, may be affected almost imperceptibly in all its manifestations, for it must be invested with an activity essentially opposed to its own natural or normal activity, in which incoherency becomes its type, before insanity positively declares itself. How many individuals who cannot be accused of being really insane are all their lives distinguished from other people by the *bizarrerie* of their character, by their excessive levity, restlessness, and the versatility of their ideas. At one time they are passionate, at another gentle; now violent, then pusillanimous; at one time they are elated by gaiety, at another oppressed with their own moroseness. And such persons, too, are often remarkable for the activity of their intellect, the extent of their understanding, and its precocious development; or it may be the reverse of all this; for their mind may be, *ab origine,* dull, inapt, and of an inferior order. Some in this 'mixed state' have been impelled to the study of the arts and sciences, in which they have risen and acquired eminence; others have given way to their passions and propensities, and plunged into all the extreme horrors of disgusting libertinage. Here, also, it may be remarked, and this observation deserves attention, I have repeatedly noticed that the relations of patients visiting the insane at the Charenton and other asylums, have exhibited signs of mixed reason and insanity strikingly like the disease of the persons they have come to visit. Twenty years ago, when an interne at the Charenton, how often have I been struck with the bizarre manners of those bringing an insane member of their family into the establishment; their extreme loquacity, their confused answers—sometimes diffuse, sometimes slow,—their laconic style of expression, their peculiar gestures, their demeanour, and the unusual expression of their physiognomy, which any inexperienced eye may perceive, and above all—which we should attend to particularly when they give an opinion of the mental state of the patient they present. Nay, we may observe that they often entertain the very ideas of their afflicted relatives—not that they acknowledge this to themselves, of which they are not conscious; for they emphatically condemn these very ideas, but in so doing, the *opiniatre* manner in which they discuss the case, indicates the uneasiness they are under, without their being in the least degree aware of its real cause. This observation applies not only to persons of humble birth and without education, whose position places them beyond the reach of those prejudices and false judgments, which may influence better informed minds; but also to persons in the higher classes of society. It is pathognomonic of the mixed state; not a co-existent, but an absolute fusion between insanity and reason.

"In illustration of this state, I could cite a great number of facts, but shall confine myself to the following:—Monsieur B—— brought his sister to the Charenton, to be treated for 'a disease of the nerves,' [these are his expressions,] and gave us the following account—'Mademoiselle is twenty-five years of age, her bodily health has always been good; menstruation regular; from infancy she possessed remarkable quickness of ideas, great irritability of feeling, and a susceptibility, sometimes amounting to a state of intellectual exaltation, which excited just fears respecting her health. She had a way of perceiving things rarely in accordance with the views of other persons; she formed her judgments with an unusual spirit of exaggeration and tenacity. Sensitive to the highest degree, and of versatile character, her affections and passions, kind or unkind, always exceeded rational bounds. In 1820, she was suddenly seized with an attack of mania, which lasted some days only; before which period it was impossible for her parents to decide whether she was insane or not, or to convince themselves whether her various extravagant acts were the result of mental disease, or depended upon any other unhappy influence.' Mademoiselle is still in the house, and at different times I have questioned her respecting the character and habits of the brother who conducted her to the Charenton, and that which she told us of him he had himself repeated to us. 'My brother is an unhappy fellow, desirous of doing good; generous to prodigality; I owe him much; but it is rare to find so strange a character as his; a combination of the most opposed qualities; he is not the same man two hours together; the slightest cause will put him into the most violent and unheard-of rage against the persons he most loves; but he is incapable of bearing malice, and there is nothing he would not do to efface the unhappy impression which he has made by ill temper; you must place no reliance on his words to-day, for he will have forgotten them to-morrow; attach no importance to what he says; he is so *entêté*, so opiniated, and when he sees anything in a new light, he altogether forgets the past; yet I believe him to be very discerning. He reflects little, judges quickly, and takes up a resolution with wonderful promptitude. He does everything, they say, by *inspiration.* The events of the revolution in July, 1830, gave rise, in him, to an exaltation of ideas which for a long time kept me and my sisters in great anxiety.'

"This was the narration of the sister, and I may add, that every person who has seen Monsieur B—— in his own house, has been struck with the oddity of his manner, his unceasing loquacity, his *brusque* manner, and the restlessness, or mobility of his features. During a long conversation I had with him, I expressed my surprise that he could with such incredible facility find means to explain the most unreasonable acts of his sister; and at the same time to vindicate those which bore the more obvious marks of her malady. Notwithstanding all my endeavours to persuade him that his views were wrong, I could not succeed; my efforts could not reach a conviction obviously beyond the pale of reason. When I asked him whether his sister were the only person in his family affected with insanity—'My mother,' he answered, in a tone of great carelessness, 'is mad; she has been in your house for the last five years. Madness with us is hereditary; I shall not escape it any more than my sister; and, to tell you the truth, it is very possible I feel it now.' As frequently happens upon the invasion of insanity, Monsieur B—— already felt a vague presentiment of his impending malady.

"Mademoiselle C—— has been insane for many years; and is at present in a state of profound dementia. Upon the accession of her disease, certain fixed ideas, which it was difficult to discover, rendered her taciturn and melancholic; but sometimes a paroxysm came on which rendered it necessary to use restraint. Monsieur C——, her brother, is colonel in a cavalry regiment. His habits, his solitary and retired mode of life, his unequal temper, the peculiar modes he took of taking care of his health, a nervous susceptibility which isolated him from society, &c., gave rise to a general opinion among his friends that he was in a state bordering upon insanity. Nevertheless, Monsieur C—— fulfilled all his duties with remarkable exactitude and intelligence. His bravery was, under a multitude of difficult circumstances, brilliant; no soldier in his regiment acquired, in this respect, a reputation equal to his. Yet numerous anecdotes are related of him, proving him to have exhibited, on many occasions, a high state of maniacal exaltation. These facts I had from an officer of his own corps.

"Monsieur T—— is in a state of dementia, complicated with chronic encephalitis consequent upon mania, which was accompanied with grand and ambitious ideas. The slightest contradiction put him into a rage which scarcely anything could calm, and which frequently brought on the most distressing scenes. One of his brothers, at whose instance he had been admitted as a patient into the Charenton, came, a few days afterwards, to see the director of the establishment, and to protest against his arbitrary detention. He declared that he had never shown the least sign of mental derangement. Everything which had occurred might be attributed to the odious intrigues and vexations he had endured, which perfectly explained the exasperation and furor into which he had been driven. Before addressing us, he had obtained an interview with Louis Philippe, (August, 1830,) who sent him to the police. At first sight, the habitual twitching of his face, the continual movement of his arms and legs, above all, his fixed look, augured ill of his mental state. He spoke high, with much volubility, and in a confused manner; not forgetting the principal subject, but interrupting it with innumerable digressions. He had received a good education, yet forgot the most simple courtesies of life. One of the physicians upon whom he called was so annoyed by his conduct, that he was obliged to order his servants to put him out of the house. In vain did they repeat to him that his brother was actually insane, and that his disease was complicated with symptoms which indicated that he would become incurable.

"Professor Lordat, upon the case of the famous Barthez, observes, 'His difficult temper, which makes it a punishment to all who have to attend upon him, rendered him insupportable to himself. He employed every ingenuity to make himself miserable. One day, when he was complaining of his *chienne de vie*, he was reminded of how many causes he had to be thankful for his lot; upon which he answered, 'True, but my own character renders it nugatory.' When he had sealed a letter, if the impression were not good he was out of patience half the day. It is difficult to believe, that after printing his discourse on the genius of Hippocrates, he passed an entire night in sleepless vexation, because, after casting off the first sheet, he discovered that in the first *e* of the word Génie, on the frontispiece, the superior horizontal accent was broken. Nothing annoyed him so much as to suppose that any person in any way depreciated his fame; he became irritable and distrustful, and occupied himself entirely with the *mênage* of his house. The Père de Barthez starved himself to death, in the ninetieth year of his age, after the death of his second wife!"

The preceding facts clearly prove that, under the influence of hereditary transmission, the moral faculties undergo modifications, more or less marked, which, without amounting to manifest declaration of insanity, should induce us to pause before we pronounce any opinion respecting the sanity of such persons; for although the disease be not declared, still insanity does exist, albeit, so blended with reason, that the one state cannot be clearly separated from the other.

In this kind of insanity—the mixed state—there is generally the highest degree of mental activity; nay, more, it is in the nature of this description of madness to translate itself into manifestations of high moral and intellectual superiority.

"We may easily conceive how those organic conditions which are the most favourable to a high development of the intellectual faculties are precisely those which are most likely to give rise to insanity. The accumulation of vital force in any organ (looking at the point physically) may give rise to two consequences, both of which are equally possible:—1st, an undue energy in the *functions* of the organ; and, 2ndly, a consequent *deviation* or *aberration* of its functions. One of the most convincing proofs of what is now asserted is, that when the intellect is in its highest degree of ascendancy—its *apogee*—so brilliant are its coruscations, that the philosophers of antiquity conceived that such a state of inspiration must come directly from the gods. Hence, too, it has, from the time of Plato, been held that madness and genius are so nearly allied, as for the terms to be almost synonymous. In many respects I doubt if any modern composer was ever more led away by this peculiar kind of mental *extasê* than Donizetti. The idea—the inspiration of the moment—'*l'estro*,' he used to say, took possession of him upon a sudden, quite unforeseen, and while he was in the midst of other occupations. Far from seeking such inspiration, he had rather to protect himself from it. The following anecdote we had from the *Maestro* himself—He was one day at dinner at Madame de C——'s; he joined in the conversation, and was not pre-occupied with any other idea, when gradually he became, as it were, absent, and lost to all that was passing round him. Suddenly, he rose up abruptly, addressed a few hasty words to the lady of the house, and retiring into an adjoining apartment, composed, almost in a breath, nearly an entire act of one of his last operas. Towards the close of his intellectual life, it was our lot to witness something of the same kind; it was at that period when the name of Felicien David appeared, like that of a meteor, in the musical world. 'I regret,' said Donizetti, 'not to have found in the *Désert* an air sufficiently expressive for the sailors of the Nile, in rowing.' At his request, I endeavoured to recite a few lines; scarcely had I commenced, when he suddenly interrupted me, snatched up a pen, traced rapidly some horizontal bars over a blank sheet of paper, which was soon covered with notes. He was under the influence of his Genius—the '*l'estro*,' of former years; but alas! it was too late—the power within had failed; he could not collect two ideas."

In continuation of this highly interesting subject, Dr. Moreau next directs our attention to the fact, that superior mental faculties in parents frequently communicate to their children an hereditary predisposition to madness, in different forms and degrees.

"It is a remarkable circumstance, well known," he observes, "to those who have made insanity a special study, that it is in those families the members of which are most distinguished for their intellectual qualities, that the greatest number of the insane are found. It is among them we most frequently find the two extreme contrasts of individuals, who by the inferiority or superiority of their intellectual faculties occupy almost every degree in the scale of intelligence, from idiocy—that *triste* condition over which human reason scarcely throws a redeeming ray of light—up to those sublime heights of genius where the affective faculties spring up and flourish as in their native soil. It is fourteen years ago since I pointed out this circumstance—that the families which have given to France the most illustrious men of genius, distinguished for their military and administrative accomplishments, and from among whom might be selected the highest examples of courage, devotion, and mental energy, are those which have unhappily produced the greatest number of victims cut down (*moissonnés*) by acute and chronic affections of the nervous system.

"More eminent men have been educated, or rather risen, from the Ecole Polytechnique than from any other school in Europe, yet it is a remarkable fact, that a greater number of insane patients have come out of this than out of any other institution in France. When I reminded the celebrated Dr. Charrier of this circumstance, he corroborated it by many interesting cases which had fallen under his observation. Whence does this arise? Is it to be attributed, as is generally supposed and affirmed, to the kind of studies assigned to the pupils of the Ecole Polytechnique? Not in the least degree; for in that case, we might predicate the very opposite result, because if anything can tend to preserve the integrity of thought and judgment, or discipline the mind so as to maintain it in its right path, assuredly it is the study of the exact or mathematical sciences. The truth would appear to be, that these sciences are cultivated only with advantage by those who are endowed with a highly-developed organization, which is attended with a predisposition to nervous diseases of every description. Constant assiduity, the effort required in special studies, may aggravate this predisposition, but never could create it."

There is one remark which Dr. Moreau makes, in concluding his observations, of great practical importance—viz., that aberration connected with this highly-organized intellectual condition indicates, generally, the worst description of cerebral lesion—absolute and incurable idiocy or imbecility—much more frequently than any partial perversion of the mental faculties. It is a common observation, that the offspring of men of genius are not only inferior to their parents, but, in point of capacity, below the average of their fellow men. Few in early life escape convulsive attacks, more or less severe, and cerebral symptoms, with, to a certain extent, some lesion or other of the intellectual functions. "True genius," exclaim, with one common voice, Spurzheim, Virey, Lordat, Burdach, &c., "stands alone—it is isolated;" "*il ne se réveille point dans sa postérité.*"

These various modifications of the intellectual faculties may become complicated with other pathological conditions incident to the peculiar idiosyncracy and physical constitution of particular individuals, such as neuralgic affections, epilepsy, paralysis, &c.; but we have already exceeded our limits. The most interesting portion of "*le Chapitre Oublié*" is that which relates to the "*état mixte*," a form of insanity which we have ourselves constantly met with in practice, and which must come daily under the observation of all who are engaged in this department of the profession. We regret, however, to say, that Dr. Moreau has by no means clearly substantiated and elucidated the necessity of our acknowledging this nosological distinction; he tells us that the "*état mixte*" admits of a clear diagnosis, and superinduces distinct pathological appearances, and when we expect that he is about to prove logically his very first proposition, he starts off into a disquisition upon the laws of hereditary transmission, leaving us to solve for ourselves the problem he has enunciated. He leads us to the entrance of the path, and leaves us to explore it for ourselves—nay, had we not been already familiar with this form of the disease, we should not have perceived the application which he might, and which *we* perhaps have, made of his views.

The "mixed form of insanity" may be clearly defined: it is attended with peculiar symptoms; it admits of a specific plan of treatment, and its pathological results are of a distinct and characteristic nature. We shall on a future occasion enter more fully into this subject.

